# Periaortitis and diffuse subendocardial vasculitis in a patient with systemic lupus erythematosus – Lupus flare or a coexisting disease?

**DOI:** 10.31138/mjr.31.2.216

**Published:** 2019-12-15

**Authors:** Ourania Gioti, Evangelos Theotikos, Argyro Lazarini, Andreas S. Triantafyllis, Alexia Balanika, Maria Boutsikou, Pavlos Tsinivizov, Antonis Fanouriakis, Antonia Elezoglou

**Affiliations:** 1 Department of Rheumatology, Asklepieion General Hospital, Athens, Greece; 2 Department of Cardiology, Asklepieion General Hospital, Athens, Greece; 3 Department of Radiology, Asklepieion General Hospital, Athens, Greece; 4 Cardiac MRI Unit, Mediterraneo Hospital, Athens, Greece

**Keywords:** Systemic lupus erythematosus, antiphospholipid syndrome, diffuse subendocardial vasculitis, periaortitis

## Abstract

Systemic lupus erythematosus (SLE) is a heterogeneous disease with a broad spectrum of clinical manifestations. Periaortitis is a rare disorder which may manifest isolated or in association with other autoimmune diseases, including SLE. Another rare, yet severe cardiovascular manifestation of lupus is diffuse subendocardial vasculitis (DSV), which should be suspected in patients presenting with myocardial hypokinesis, impaired ejection fraction and normal coronary angiography. Cardiovascular Magnetic Resonance (CMR) imaging is crucial to distinguish between DSV and lupus myocarditis, which should also be included in the differential diagnosis.

Herein, we describe a case of a female patient with pre-existing SLE, who presented with both periaortitis and DSV, and discuss the diagnostic challenges associated with these rare manifestations.

## INTRODUCTION

Systemic lupus erythematosus (SLE) is a multisystem autoimmune disorder with protean clinical manifestations, involving almost all organ systems. Cardiovascular manifestations of SLE include pericarditis, premature atherosclerosis, valvular disease and, less commonly, myocardial dysfunction. The latter, in the context of myocarditis, typically occurs in the presence of generalized disease activity. Diffuse subendocardial vasculitis (DSV) is a less frequent entity affecting the myocardium, recently identified by cardiovascular magnetic resonance (CMR).^1^ It can lead to severe heart failure and is associated with high mortality and morbidity.^2^ Another rare manifestation occasionally associated with autoimmune diseases (including SLE and antiphospholipid syndrome [APS]) is periaortitis, a fibro-inflammatory condition that affects mainly the abdominal aorta and its branches.^3^

We herein present a patient with a history of SLE in long-standing quiescence, who presented with an acute inflammatory syndrome, including both periaortitis and DSV, and discuss the relevant diagnostic challenges.

## CASE DESCRIPTION

A 44-year-old woman presented to the emergency room (ER) complaining of low back- and abdominal pain lasting 3 days, accompanied by an episode of vomiting. Her past medical history included a diagnosis of SLE without major organ involvement (digital vasculitis, arthritis and compatible serology [positive antinuclear antibodies, increased anti-double-stranded DNA antibodies and low levels of complement C3, C4]). She also carried a diagnosis of obstetric APS, based on a history of a 2^nd^ trimester foetal loss and triple antiphospholipid antibody (aPL) positivity. Her treatment included low-dose aspirin 100mg, azathioprine 50mg, hydroxychloroquine (HCQ) 200mg and prednisolone 5mg.

In the ER, the patient was afebrile yet ill-appearing, with mild tachycardia (103 beats/minute) and normal blood pressure and oxygen saturation (97%). Physical examination revealed epigastric and right hypochondriac tenderness on palpation. Laboratory tests showed an increased white blood count of 18500 cells/μL, predominantly neutrophilic (85%). Serum C-reactive protein (CRP) and erythrocyte sedimentation rate (ESR) were profoundly elevated, 350 mg/l (70 times the upper normal limit) and 110 mmHg (normal range 0–20), respectively. Chest X-ray, urinalysis and electrocardiography (ECG) were un-remarkable. The patient underwent an urgent abdominal CT scan, which revealed a low-density soft tissue mass surrounding the abdominal aorta and extending to involve the renal arteries, findings suggestive of periaortitis. The patient was admitted to the Rheumatology Department for further work-up and treatment. Immunologic tests revealed positive aPL, negative anti-dsDNA and reduced complement levels (C4 0.02 gr/l, normal range 0.1–0.4). As IgG4-related disease (IgG4-RD) was also suspected, serum IgG4 was ordered and was found low (0.8 mg/dl, normal range 8–140). Anti-neutrophil cytoplasmic antibodies (ANCAs) were also negative.

Prednisolone 50mg was administered intravenously, followed by 1gr methylprednisolone the next day, with immediate, remarkable clinical response. Later during the second day of hospitalisation, though, the patient complained of dizziness and mild chest pain. There was an increase in the levels of serum aspartate aminotransferase (AST), creatine phosphokinase (CPK), creatine kinase-MB (CK-MB) and troponin, the latter reaching a peak of 35000pg/ml (normal range < 15 pg/ml) during hospitalization. ECG findings were similar to ECG of admission, while heart echocardiography revealed diffuse hypokinesis of the inferior, lateral and posterior wall and an impaired left ventricular ejection fraction (EF) of 40%. After cardiology consultation, and to rule out an acute coronary syndrome, the patient underwent coronary angiography, which revealed normal coronaries. Chest CT and aortography revealed a normal-appearing thoracic aorta, but additionally showed bilateral scattered lung infiltrates and areas of ground glass involving all lung areas. The findings were attributed to pneumonitis, despite the patient having no respiratory symptoms or signs.

**Figure 1.**
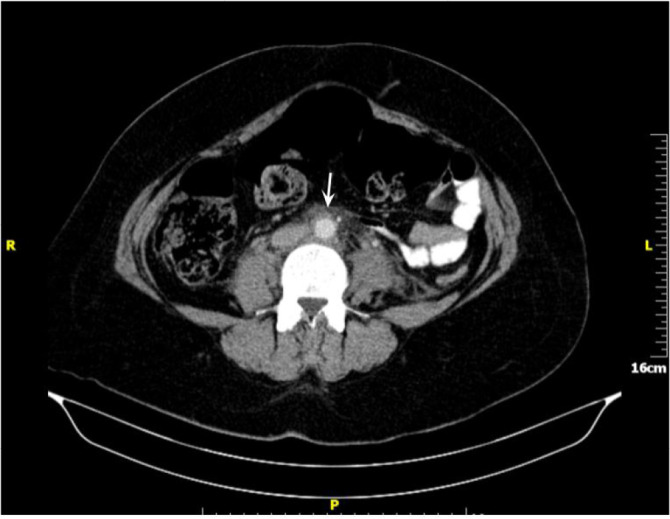
CT findings in periaortitis, low-density soft tissue around the abdominal aorta.

**Figure 2.**
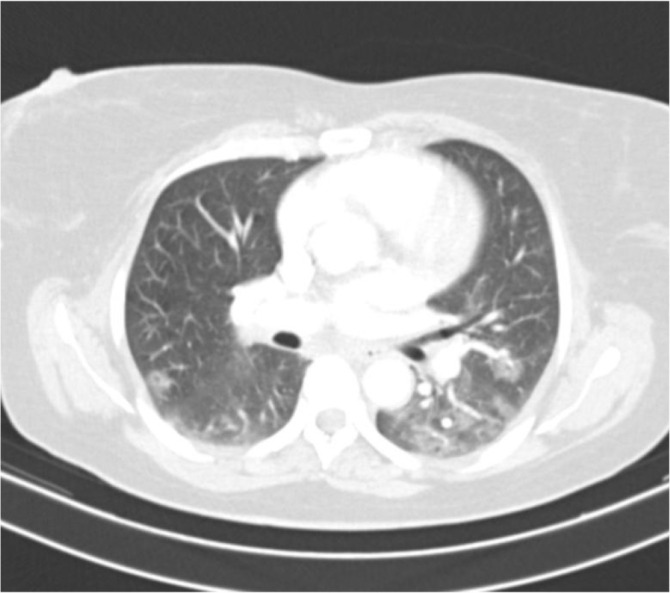
Scattered bilateral lung infiltrates and areas of ground glass.

With diffuse myocardial hypokinesis and a normal coronary angiogram, a diagnosis of myocarditis was suspected; to this end, CMR was performed. In spite of the absence of oedema in STIR T2-weighted images, late gadolinium enhancement (LGE) images revealed a diffuse subendocardial enhancement regarding the posterior, inferior and lateral wall, without anatomic distribution of a coronary artery. These findings (diffuse subendocardial enhancement in LGE images) were compatible with diffuse subendocardial vasculitis (DSV).

In the absence of a satisfactory alternative diagnosis (IgG4-related disease, ANCA or other vasculitis), a diagnosis of a SLE flare with rare manifestations, including periaortitis, pneumonitis and DSV was presumed. Three daily pulses of methylprednisolone (1gr) were administered, followed by 0.7 mg/kg/day prednisone orally, together with pulse cyclophosphamide (CY 0.75 mg/m^2^), as in organ-threatening SLE. Symptomatic treatment for congestive heart failure, including carvedilol 12.5 mg/day and ramipril 2.5 mg/day was also initiated. The patient’s symptoms improved rapidly, accompanied by significant improvement of laboratory and imaging findings; levels of troponin and CRP gradually normalised and the EF improved to 48%. Over the 3-month follow-up, the patient remains clinically stable, while on monthly pulse CY therapy and tapering prednisone doses. At last heart echocardiography, EF has raised to 58%. A follow-up CMR is scheduled in 3 months, to be compared with baseline findings.

**Figure 3.**
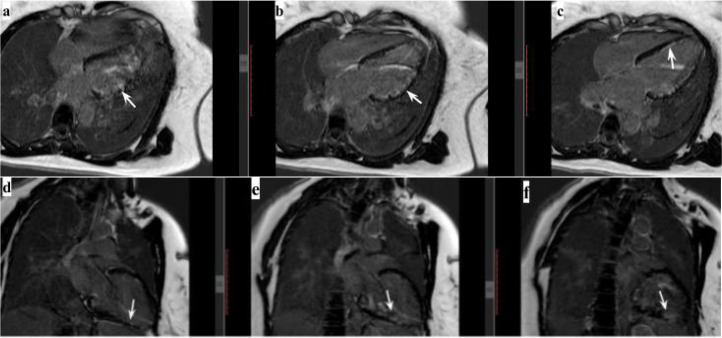
CMR, diffuse subendocardial enhancement in LGE images regarding the lateral wall (a,b), the ventricular septum (c) and the inferior wall (d,e,f).

## DISCUSSION

We describe a female patient, with a history of SLE and obstetric APS, who presented with three rare, albeit major manifestations, namely periaortitis, DSV and pneumonitis. Ultimately, these were all attributed to a SLE flare, as we were not able to justify an alternative diagnosis.

Traditionally, very high CRP levels are rarely attributed to increased SLE activity.^4^ Indeed, CRP increases slightly in patients with a disease flare and it has been proposed by some to serve as a marker to differentiate disease activity from infection.^5^ An extensive work-up to rule out infection was performed in our patient, and it could not be established. On the other hand, as periaortitis is considered a highly inflammatory condition, the markedly increased CRP levels could be associated with this entity. In idiopathic periaortitis, acute-phase reactants (ESR and CRP) are typically elevated and usually decline to normal values following therapy initiation, though they are not always reliable in monitoring disease activity.^6^ Thus, imaging studies (CT or MRI) are necessary during follow up.^7^ The inflammatory markers in our case normalised following immunosuppressive therapy; a CT scan is additionally planned to monitor the periaortic mass.

Periaortitis is characterised by the presence of a fibro-inflammatory tissue, usually surrounding the infra-renal part of the abdominal aorta. It may occur isolated or in the context of systemic autoimmune diseases, such as SLE,^3^ while there are only two case reports which associate periaortitis with APS.^8,9^ It is also typically included in the spectrum of IgG4-related disease (IgG4-RD). Periaortitis may also be secondary to malignancies, infections or certain drugs. Regarding infectious causes, tuberculosis, actinomycosis and histoplasmosis should be considered. It is commonly the result of local spread of a contiguous infection focus, for example spinal or paraspinal abscesses.^3^

A biopsy is performed in cases with clinical or imaging findings suggestive of secondary forms, especially underlying malignancy. Some CT features (atypical location, bulkier appearance, anterior displacement of the aorta, mass effect on adjacent structures) help to suspect a malignant process.^7^ However, the sensitivity and specificity of these characteristics are poor. Moreover, a biopsy may contribute to support the diagnosis of IgG4-RD, as low levels of serum IgG4 do not exclude this diagnosis.^10^ In our case, given the difficulty in performing a biopsy, together with the absence of findings suggestive of underlying malignancy and the immediate response in therapy, we decided not to have the patient undergo this procedure.

Pathophysiologically, periaortitis was originally considered as a result of a localised reaction to atherosclerosis. However, subsequent studies based on histopathological and genetic data have supported the hypothesis of a systemic immune-mediated process.^11,12^ In our patient, the coexistence of SLE and APS, rapid response to immunosuppressive treatment and absence of atherosclerosis pointed towards an inflammatory/autoimmune manifestation.

DSV is a recently characterized entity, which can develop in the context of autoimmune diseases, like SLE, systemic sclerosis, rheumatoid arthritis and sarcoidosis.^1,13^ It is usually characterized by severely compromised left ventricular (LV) function.^2^ CMR is considered crucial for its diagnosis, as it can differentiate between DSV and other conditions, such as infarction or myocarditis.^14^ More precisely, regarding the LGE images, subendocardial/transmural enhancement in the distribution of a coronary artery is compatible with myocardial infarction. One the other hand, subepicardial/intramural enhancement and diffuse subendocardial enhancement are suggestive of myocarditis and DSV, respectively. Furthermore, CMR can help to distinguish between acute (oedema in T2/STIR images and diffuse subendocardial fibrosis [DSF] in LGE images) and chronic DSV (DSF in LGE images, without oedema in T2/STIR), a differentiation with important therapeutic implications.^2^ In our case, CMR revealed a diffuse subendocardial enhancement in the LGE images, without oedema in the T2/STIR images, findings more compatible with chronic DSV. However, considering that the patient was experiencing an acute cardiac syndrome (symptomatic, with constantly increasing troponin levels) in the presence of a generalized autoimmune flare, a diagnosis of acute DSV was presumed. Initiation of combined immunosuppressive and symptomatic treatment led to rapid symptom control.

In conclusion, we describe a case of a woman with a history of SLE presenting with periaortitis and DSV as major manifestations of a lupus flare. The patient showed significant and rapid improvement after initiation of immunosuppressive therapy. Albeit rare, these conditions should be included in the differential diagnosis in patients with SLE, when clinical presentation combined with laboratory and imaging studies are indicative.
